# Modular design and development methodology for robotic multi-axis F/M sensors

**DOI:** 10.1038/srep24689

**Published:** 2016-04-22

**Authors:** Qiao-Kang Liang, Dan Zhang, Gianmarc Coppola, Wan-Neng Wu, Kun-Lin Zou, Yao-Nan Wang, Wei Sun, Yun-Jian Ge, Yu Ge

**Affiliations:** 1 College of Electric and Information Technology, Hunan University, Changsha, Hunan 410082, China; 2 State Key Laboratory of Advanced Design and Manufacturing for Vehicle Body, Hunan University, Changsha 410082, Hunan, China; 3 Department of Mechanical Engineering, York University, Toronto, ON M3J 1P3, Canada; 4 National Engineering Research Lab for Robot Vision Perception and Control, Hunan University, Changsha, Hunan 410082, China; 5 Institute of Intelligent Machines, Chinese Academy of Science, Hefei, Anhui 230031, China

## Abstract

Accurate Force/Moment (F/M) measurements are required in many applications, and multi-axis F/M sensors have been utilized a wide variety of robotic systems since 1970s. A multi-axis F/M sensor is capable of measuring multiple components of force terms along *x*-, *y*-, *z*-axis (*F*_*x*_, *F*_*y*_, *F*_*z*_), and the moments terms about *x*-, *y*- and *z*-axis (*M*_*x*_, *M*_*y*_ and *M*_*z*_) simultaneously. In this manuscript, we describe experimental and theoretical approaches for using modular Elastic Elements (EE) to efficiently achieve multi-axis, high-performance F/M sensors. Specifically, the proposed approach employs combinations of simple modular elements (e.g. lamella and diaphragm) in monolithic constructions to develop various multi-axis F/M sensors. Models of multi-axis F/M sensors are established, and the experimental results indicate that the new approach could be widely used for development of multi-axis F/M sensors for many other different applications.

Touch and vision are the most important perceptions for a person or a robot while assessing and exploring an object with their hands or manipulators^1^,^2^,^3^. Nowadays, multi-axis Force/Moment (F/M) Sensing technology is one of the primary used means of interaction with environment for robotic systems. Even so, most developed F/M sensors detect F/M (from physical six degrees of freedom) in less than three axial directions. More recently, high-performance F/M sensors that can detect multiple components of applied load have promising robotic applications, especially in robotic systems to achieve reliable and dexterous manipulation^4^,^5^. Among the various methods to measure F/M, sensors based on resistive sensing principle have been widely investigated due to their simple construction, compatibility with VLSI (Very Large Scale Integration), and convenient availability of the output signal. Also, there are advantages of resistive sensors; high reliability, easy fabrication process, and adjustable resolution. These advantages are over other measuring techniques (e.g. capacitive^6^, piezoresistive^7^, inductive^8^, optoelectronic^9^, piezoelectric^10^ and etc.) and illustrate tremendous promise for the detection of multi-axis F/M^11^,^12^,^13^. The EE (Elastic Element), the most critical mechanical component of a multi-axis F/M sensor, serves as the reaction mechanism to the applied load by its deformation or strain. The appropriate design of an EE is always the consequence of a trade-off among various objectives such as sensitivity, stiffness, repeatability, and linear performance. For example, an F/M sensor with high global sensitivity is always characterized by a flexible EE structure, which also results in a low stiffness of the sensor. Recently, there has been a growing interest in the exploration of the EE design. Several design criteria should be considered when designing a certain EE as following^14^,^15^: i. High sensitivity and stiffness. ii. Simple structure with minimum volume and especially reduced height. iii. Measurement isotropy and low coupling effects among components. iv. Small error due to nonlinearity, hysteresis and repeatability, etc.

Although there have been a number of approaches to design and develop multi-axis F/M sensors, which have been proven to be successful in detecting multi-axis F/M, the vast majority of prior designs have relied on the experience of designers or direct optimization algorithms. However, more and more high-performance F/M sensors adopt novel EE structures with complicated geometric shapes. Consequently, classical closed-form analysis and direct optimization approaches are difficult and even impossible to obtain and implement.

Traditionally, individual design and development approaches of multi-axis F/M sensors have varied structures and configurations. This means that the structures and geometry dimensions of the EEs are always determined according to the specific measurement range, number of components, performance requirements. Therefore, the development of an efficient design and fabrication method for multi-axis high-performance F/M sensors in a simple and systematic manner is still a challenge the scientific and engineering communities^16^.

In this study, we demonstrate a new approach to the design and development of a series of multi-axis F/M sensors with different number of components. Moreover, the main contribution of this paper lies in the following aspects: (i) efficiently designing a series of multi-axis F/M sensors with simple modular elements; (ii) scientifically determining the optimal structural parameters of the modular elements in order to solve the trade-off among the different performances. Consequently, 4-axis, 5-axis and 6-axis F/M sensor prototypes are deigned and fabricated. The results of calibration experiments show that the proposed approach is systematic, universal and can be standardized for multi-axis F/M sensors. This is the first report on the standard and methodical design of multi-axis F/M sensors with modular EEs.

## Results

### Structure and characterization of the modular EEs

**Figure 1:**
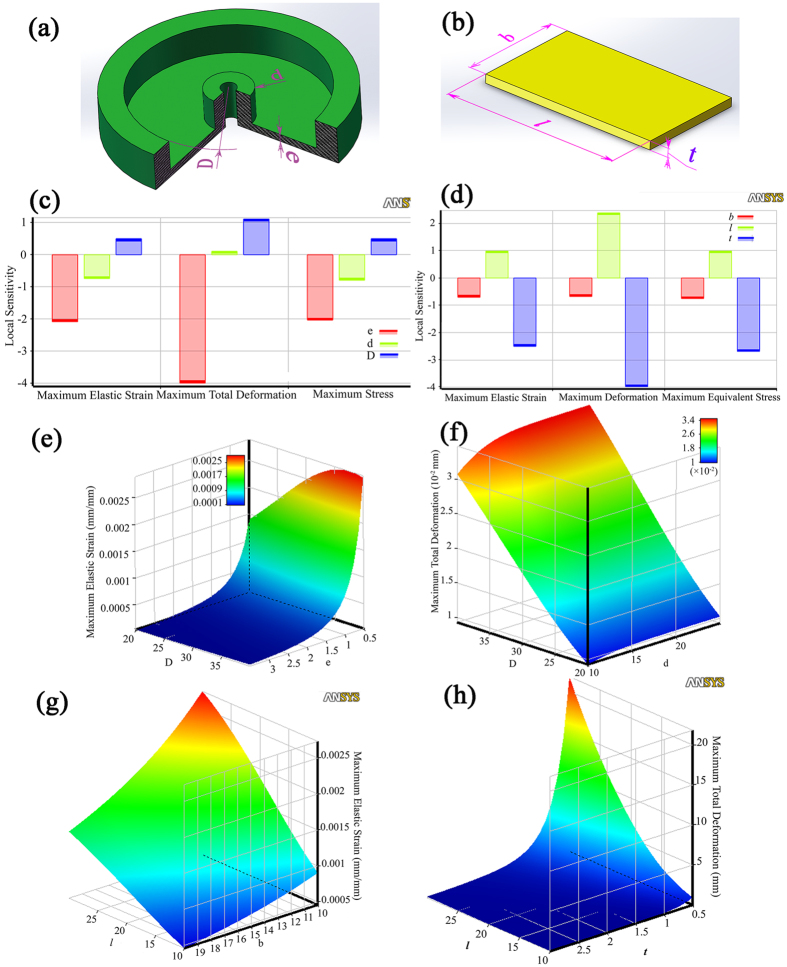
Structure and characterization of the modular EEs. (**a**) Structure of the circular diaphragm. (**b**) Structure of the cantilever beams. Sensitivities of the input parameters of the circular diaphragm (**c**) and cantilever beams (**d**) are illustrated for comparison. Relationships between design parameters and the variation of the output variables: Maximum Total Deformation of circular diaphragm vs parameter *D* and *d* (**e**); Maximum Elastic strain of circular diaphragm vs parameter *D* and *e* (**f**); Maximum Total Deformation of cantilever beam vs parameter *l* and *t* (**g**); Maximum Elastic Strain of cantilever beam vs parameter *l* and *b* (**h**).

**Figure 2:**
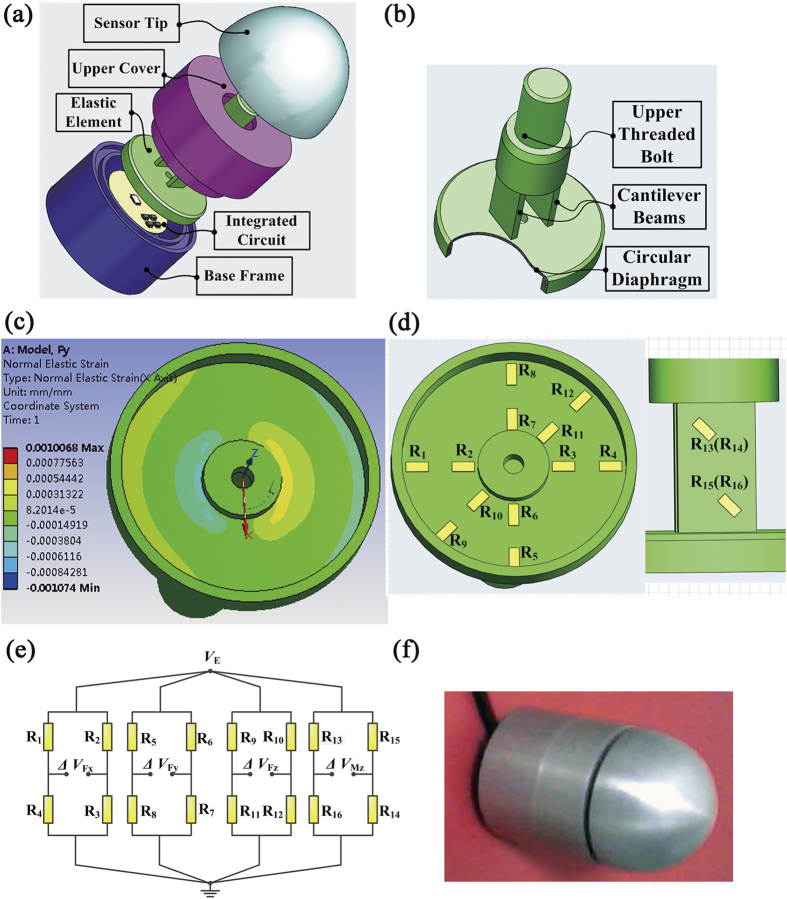
Four-axis F/M sensor with modular EEs. (**a**) Structure of the four-axis F/M sensor. (**b**) EE structure of the four-axis F/M sensor. (**c**) FEA results of strain distribution. (**d**) Arrangement strategy of the strain gauges. (**e**) Four full-bridge circuits. (**f**) Prototype of the four-axis F/M sensor.

Unlike sophisticated EE structures adopted by the majority of the existing multi-component F/T sensors, two simple elements, as shown in Fig. 1, are proposed to create a novel modular EE. A circular diaphragm (as depicted in Fig. 1a) can serve as an active sensing portion to measure the normal force (*F*_*z*_), both tangential force terms (*F*_*x*_ and *F*_*y*_), and the moment terms about the *X*-axis and *Y*-axis (*M*_*x*_ and *M*_*y*_) simultaneously. The cantilever beams (Fig. 1b) are designed to exhibit substantial flexibility in the extending direction and thus serve as active sensing segment to detect the bending moment component. Among the Fig. 1a, the structural parameters *e, d* and *D* respectively denote the thickness, inner diameter and external diameter of the circular diaphragm. And the parameters *b, l, t* shown in the Fig. 2b represent the width, length and thickness of the cantilever beam, respectively.

### Simulation-Driven Design and Optimization of the F/M sensor

The appropriate dimensions of the circular diaphragm and the cantilever beams should be the consequence of a trade-off between sensitivity, stiffness, linearity and repeatability. Here, the Simulation-Driven Design and Optimization (SDDO), which allows accelerating the process of the structure development from the conceptual design to the last product, is adopted. Based on the approach of Design Exploration provided by software ANSYS, Finite Element Analysis (FEA) and multi-objective optimization are performed^17^. Therefore, the geometry dimensions (*e, d, D* and *b, l, t*) are respectively set as the design parameters (input parameters), while the equivalent stress, normal elastic strain, deformation and the first response frequency occurred on the sensing elements are set as response parameters (output variables). Among the response parameters, the maximum equivalent stress and maximum normal elastic strain can make sure the sensor work within the elastic limit and have enough elastic strain to sense the applied forces or moments. While the maximum deformation is to ensure the sensor has a good linearity, repeatability and stiffness. And the first response frequency can enable the sensor possess an appropriate dynamic character.

Figure 1c,d, obtained from the SDDO method, illustrate the impact of each input parameter on the output variables for circular diaphragm and cantilever beam, in which a greater positive sensitivity indicates a greater impact on the output parameter value, while more negative sensitivity indicates a greater negative impact on the output value. It can be observed that the most significant input parameter to the output variables are the thickness of the circular membrane and the extending length of the cantilever beam. Figure 1e–h show the relationships between design parameters and the variation of the output variables, which is helpful to identify and understand specific changes to meet the corresponding requirements for the multi-axis F/M sensors.

## Four-axis F/M sensor

By adopting a circular diaphragm and two cantilever beams, the EE structure for a four-axis F/M sensor is proposed and is shown in Fig. 2a. The geometrical dimensions of the diaphragm and beams are determined via SDDO. The whole sensor has a size of Φ 30 mm × 35 mm as illustrated in Fig. 2b, and it consists of a base frame, an EE, an upper cover, an integrated circuit and a sensor tip. Once the sensor is mounted onto a robot fingertip, it can measure the normal force (*F*_*z*_), both tangential force terms (*F*_*x*_, *F*_*y*_), and the moment term about *z*-axis, simultaneously. The measurement ranges of this sensor are set to *F*_*x*_ = *F*_*y*_ = [−30 N, +30 N], *F*_*z*_ = [0, 50 N] and *M*_*z*_ = [−8 Nm, +8 Nm]. The strain distributions on the sensing portions of the EE, as shown in Fig. 2c, is generated by the static structural analysis via ANSYS. The inner and outer rings of the circular diaphragm as well as the area near the diagonal of cantilever beams where maximum elastic stain occurs are selected to bond the strain gauges. In accordance with the strain distribution patterns, strain gauges are bonded onto the circular diaphragm and cantilever beams in specific locations and orientations, as shown in Fig. 2d. All of the bonded strain gauges are connected electronically to form four separate full-bridge circuits as shown in Fig. 2e. A prototype of the four-axis F/M sensor designed with optimal parameters via SDDO has been constructed, as shown in Fig. 2f. Next, through a calibration experiment, we collected the contrastive sample data between the output of the sensor and the actual applied loads recorded by the sensor. After a neural network decoupling method and calculation, the maximum nonlinearity error and maximum coupling error of the F/M measurement can be reached at 0.5% F.S. and 1.5% F.S., respectively. In this paper, all the errors of the F/M sensor are calculated according to the formula 
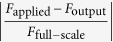
, where the *F*_applied_ is the force/torque value actually applied along each direction of the force sensor, *F*_output_ is the output force/torque value of the sensor and the *F*_full-scale_ is the maximum force/torque in each direction of the sensor.

According to the above formula, when the force is applied along the *F*_*x*_ direction for example, the error rate on all the six direction can be calculated through the formula. Among them, the error rate on *F*_*x*_ direction is called as I-type relative error (namely the nonlinear error), which reflects the deviation degree of the predict results compared with the actual applied value. However, the error rates along the other five directions are named as II-type relative errors (namely the coupling errors), which mirror the decoupling degree between each dimension.

## Five-axis F/M sensor

**Figure 3:**
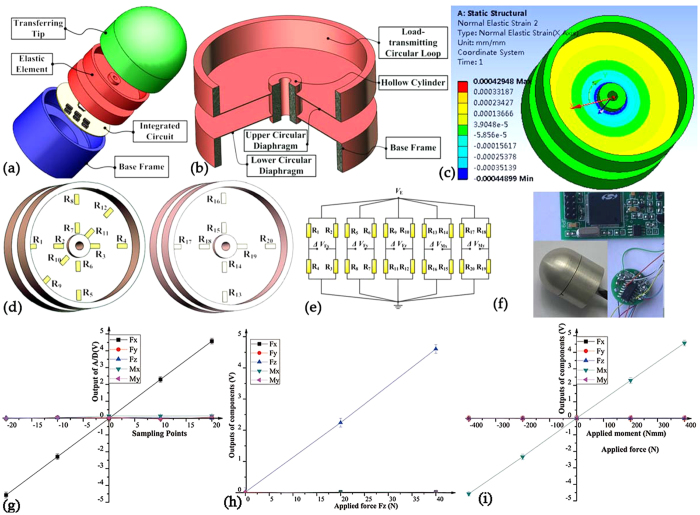
Five-axis F/M sensor with modular EEs. (**a**) Structure of the five-axis F/M sensor. (**b**) EE structure of the five-axis F/M sensor. (**c**) FEA results of strain distribution. (**d**) Arrangement strategy of the strain gauges. (**e**) Five full-bridge circuits. (**f**) Prototype of the five-axis F/M sensor. (**g**) Experimental result of component *F*_x_ (similar with *F*_y_). (**h**) Experimental result of component *F*_z_. (**i**) Experimental result of component *M*_x_ (similar with *M*_y_).

A five-axis F/M sensor can be applied to a robot hand (or end effector) to measure forces along the *x*-, *y*- and *z*-axes (*F*_*x*_, *F*_*y*_ and *F*_*z*_) as well as moments about the *x*- and *y*-axes (*M*_*x*_ and *M*_*y*_) simultaneously. As shown in Fig. 3a, the proposed five-axis F/M sensor consists of a transferring tip, a rotationally symmetric EE structure with double circular diaphragms, an integrated electric circuit and a base frame. More specifically, the upper diaphragm of the monolithic EE (Fig. 3b) that serves as an active sensing portion is sensitive to the moments about the *x*- and *y*-axes (*M*_*x*_ and *M*_*y*_). Whereas, the lower diaphragm that severs as an active sensing portion is sensitive to the forces along the *x*-, *y*- and *z*-axes (*F*_*x*_ *F*_*y*_ and *F*_*z*_). The geometrical dimensions of the EE are determined via SDDO. The whole sensor has a general dimension of Φ24 mm × 30 mm, with measurement ranges of *F*_*x*_ = *F*_*y*_ = [−30 N, +30 N] *F*_*z*_ = [0, 50 N] and *M*_*x*_ = *M*_*z*_ = [−6 Nm, +6 Nm]. FEA is performed to understand the static characteristics such as the strain distribution and deformation shape. As shown in Fig. 3c, the elastic strain mainly occurs on the double circular diaphragms, and it reaches its maximum and minimum values close to the inner and outer circumferences, respectively. 20 silicon strain gauges are cemented in these locations (Fig. 3d) and connected electrically to form five separate full-bridge circuits as shown in Fig. 3e. One of the most troublesome restrictions of this type structure is the high coupling interference between the components *F*_*x*_ and *M*_*y*_, as well as the components *F*_*y*_ and *M*_*x*_. This coupling is due to the phenomenon in which the lower diaphragm has identical strain distributions (in terms of both pattern and magnitude) when loads are applied in each of the normal force *F*_*z*_, moment *M*_*x*_ and *M*_*y*_ directions. A prototype of the five-axis F/M sensor is shown in Fig. 3f. However, the cross-axis coupling has been reduced and the performance has been improved thanks to a specific static decoupling and calibration method. The calibration experiments can supply us with the corresponding sample data between the output voltage signal and the actual applied F/M value of the sensor. And the static decoupling algorithm, the neural network for instance, can help us find the mapping relationship between the input and output of the sensor, which is benefit to largely increase the measurement accuracy. As shown in the Fig. 3g–i, the experimental results show that the maximum nonlinearity error and maximum interference error are 0.14% F.S and 1.5% F.S., respectively.

## Six-axis F/M sensor

**Figure 4:**
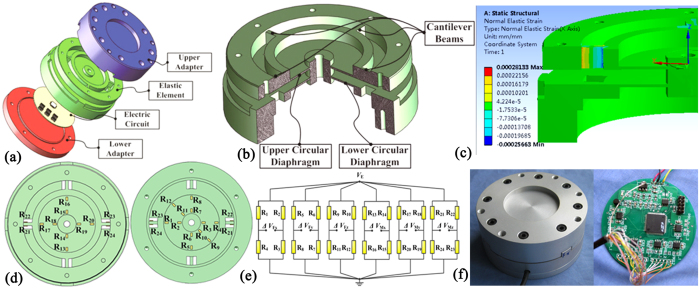
Six-axis F/M sensor with modular EEs. (**a**) Structure of the six-axis F/M sensor. (**b**) EE structure of the six-axis F/M sensor. (**c**) FEA results of strain distribution. (**d**) Arrangement strategy of the strain gauges. (**e**) Six full-bridge circuits. (**f**) Photograph of the six-axis F/M sensor prototype.

Six-axis F/M sensors are capable of sensing three components of time-varying forces (*F*_*x*_, *F*_*y*_ and *F*_*z*_) and three components of moments (*M*_*x*_, *M*_*y*_ and *M*_*z*_) simultaneously at a single point or on a distributed array of points on a surface. The proposed six-axis F/M sensor, as illustrated in Fig. 4a, consists of an upper adapter, a lower adapter, an EE and an integrated electric circuit. A monolithic EE with double circular diaphragms and four cantilever beams is presented in Fig. 4b. Practically, the upper diaphragm that serves as an active sensing portion is sensitive to the moment about the *x*-axis and *y*-axis (*M*_*x*_, *M*_*y*_), and the lower diaphragm that serves as an active sensing portion is sensitive to the normal force (*F*_*z*_) and both tangential force terms (*F*_*x*_, *F*_*y*_). On the other hand, the lamellas whose axes are perpendicular to each other in a cross-shape are sensitive to the moment about the normal axis (*M*_*z*_). The key geometrical dimensions of the sensing portions are determined via SDDO, and the entire size of the sensor is Φ 80 mm × 42 mm. The measurement range of the system is set to 0 ~ 250 N for cutting force in normal direction, ±200 N for cutting force in shear direction, and ±10 Nm for cutting moments around normal axis, and ±8 Nm for cutting moments around shear axis. As illustrated in Fig. 4c, the maximum and minimum strain of the EE take place at the ends of the beams when under the applied moment *M*_*z*_. At the same time, the elastic strain mainly occurs on the inner ring and the external ring on the circular diaphragms when under any other applied load. Therefore, these positions are ideal for strain gauges in load measurement and are oriented properly as shown in Fig. 4d. 24 bonded strain gauges are interconnected to form 6 Wheatstone bridges as shown in Fig. 4e, and their output voltage variations are proportional the small resistance changes of gauges that represent the applied loads. A prototype of a six-axis F/M sensor is shown in Fig. 4f. The results of the calibration experiment indicate that the maximum nonlinearity error and maximum interference error of the proposed six-axis sensor are 0.17% F.S. and 1.6% F.S., respectively.

## Discussion

Motivated by the importance of having an efficient way to design and develop multi-axis F/M sensors with a different number of components for various robotic applications, we have proposed a new methodology encompassed by modular EEs, i.e. circular diaphragm and cantilever beam. Additionally, the optimum geometrical dimensions of the EE are suggested to obtain via DOE method, and the strain gauges are placed at the critical points on the EE in order to obtain the maximum sensitivity and repeatability. Currently, there are many commercial three-axis and six-axis F/M sensors with acceptable performance, while other types of multi-axis F/M sensors such as two-axis, four-axis and five-axis F/M sensors are mainly designed and developed by scholars and researchers for specific robotic applications. Typically, researchers tend to utilize six-axis F/M sensors to acquire multi-axis (less than 6) F/M information. This results not only in hardware waste but also in high system error due to coupled interferences among six components. Additionally, multi-axis F/M sensors with particular requirements of its measurement range and other performance indexes need to be designed and developed individually.

With modular EEs, multi-axis F/M sensors with a different number of components, measurement ranges, and other performance indexes can be rapidly designed and developed. Moreover, by the implementation of SDDO, relationships between the design parameters and the output variables can be obtained. This is very helpful to determine key geometrical dimensions of the modular EEs. With a data acquisition system that consists of necessary hardware and software, the sensor could measure the multi-axis forces and moments simultaneously.

This work is the first to experimentally realize a modular F/M sensor design for multi-axis F/M sensors. Three cases were included to illustrate the methodology, and the results show that the maximum nonlinearity error and maximum interference error are 0.5% F.S. and 1.6% F.S, respectively. These results may also further help us understand questions such as how an EE structure affects the performance of the multi-axis F/M sensor and how to design suitable load sensing system.

## Methods

When a load is applied to the multi-axis F/M sensor, resistances change of strain gauges bonded onto the EE Δ*R*_*i*_ = *Gε*_*i*_*R*_*i*_ is sensitive to the occurred strain *ε*_*i*_, which is proportional to the applied load 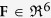, *G* and *R*_*i*_ are the gauge factor and the electrical resistance of the *i*th strain gauge.

The relation between the output voltage variation of the measuring bridges 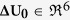 and the strains due to the applied load is given by


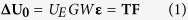


where *U*_*E*_ is the voltage excitation source of the bridges, 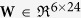 and 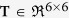 represent the bridge transformation matrix and the transducer matrix, respectively. These matrixes depend on the structure of the EE, the geometrical dimensions of the active sensing portions, the configuration of the measuring bridges, and the particular locations of the strain gauges. The applied cutting load can be determined and calculated:


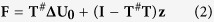


where **T**^#^ is the generalized inversion of **T**. **z** is an arbitrary 6 × 1 vector, and usually set as **z** = **O**.

To avoid the unit inconsistency problem, the transducer matrix is normalized as


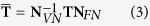


with normalization compensations


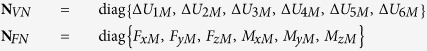


where Δ*V*_*iM*_ and *F*_*iM*_ (or *M*_*iM*_) are maximal voltage variation of the *i*^th^ measuring bridge and pre-specified maximal cutting forces (or moments).

The anisotropy index of the sensor can be obtained by the condition number of 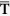
18


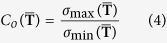


where *σ*_max_ and *σ*_min_ represent the largest and the smallest singular values of 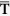, respectively.

The absolute sensitivity of the sensor can be evaluated by^19^


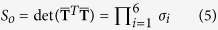


The obtained cutting load is respect to the sensor’s coordinate frame {**S**}, and it should be transformed to the tool’s coordinate frame {**T**} as


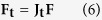


where


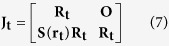


In the above relation, **r**_**t**_ is the location vector of the frame {**S**} with respect to the frame {**T**}, **R**_**t**_ is the is the orientation matrix of frame {**S**} relative to the frame {**T**}, **O** and **S** (*) are the null matrix and skew-symmetric operator, respectively.

## Additional Information

**How to cite this article**: Liang, Q.-K. *et al*. Modular design and development methodology for robotic multi-axis F/M sensors. *Sci. Rep.* **6**, 24689; doi: 10.1038/srep24689 (2016).
